# Diabetic Striatopathy: Parenchymal Transcranial Sonography as a Supplement to Diagnosis at the Emergency Department

**DOI:** 10.3390/diagnostics12112838

**Published:** 2022-11-17

**Authors:** Massimiliano Godani, Giuseppe Lanza

**Affiliations:** 1 Department of Neurology, Sant’Andrea Civic Hospital, ASL 5 Spezzino, Via Vittorio Veneto 197, 19121 La Spezia, Italy; 2 Department of Surgery and Medical-Surgical Specialties, University of Catania, Via Santa Sofia 78, 95123 Catania, Italy; 3 Clinical Neurophysiology Research Unit, Oasi Research Institute-IRCCS, Via Conte Ruggero 73, 94018 Troina, Italy

**Keywords:** diabetic striatopathy, differential diagnosis, neuroimaging, metabolic dysfunction, emergency

## Abstract

*Background*: Diabetic striatopathy (DS) is a rare condition with a debated pathophysiology; a local metabolic dysfunction is the most likely hypothesis. We present a case of DS mimicking an acute stroke, outline a few uncommon/atypical features, and report for the first time the parenchymal transcranial sonography (pTCS) findings. *Case Report*: An 86-year-old man, treated for insulin-dependent diabetes, presented at an emergency department because of the occurrence of isolated choreo-athetotic movements in his left limbs with fluctuations in the location, frequency, and duration. The blood glucose level was 569 mg/dL. Both urgent and follow-up brain computed tomography (CT) were negative for recent lesions whereas pTCS revealed hyperechogenicity in the right lenticular nucleus. Subsequent magnetic resonance imaging (MRI) showed T1-weighted hyperintensity in the right putamen with negative diffusion-weighted imaging. The symptoms were responsive to glucose control and haloperidol administration, although they persisted during sleep. *Conclusions*: Unlike previously described cases characterized by hemichorea and/or hemiballism, our patient presented with a stroke-like onset of unilateral irregular choreo-athetotic movements. Notably, based on CT alone, it would not have been possible to distinguish DS from a stroke. In this scenario, the pTCS hyperechogenicity of the right lenticular nucleus helped to hypothesize a metabolic disorder, which was subsequently confirmed by MRI.

## 1. Introduction

The definition of diabetic striatopathy (DS)—also called hyperglycemic non-ketotic hemichorea/hemiballism [1,2]; chorea/hemichorea associated with non-ketotic hyperglycemia [3,4,5]; diabetic hemiballism/hemichorea [6]; and chorea, hyperglycemia, and basal ganglia syndrome [7]—was first adopted to indicate a relatively uncommon hyperglycemic disorder associated with chorea/ballism and a typically reversible abnormality of the basal ganglia from computed tomography (CT) or/and magnetic resonance imaging (MRI) [8]. Both chorea and ballism are defined as random, uncontrollable, and involuntary jerking movements; ballism is more proximal and of a larger amplitude than chorea.

The prevalence of DS has been estimated to be 1 in 100,000 [9]; however, this is believed to be significantly underestimated because most clinicians are not familiar with the condition and, therefore, may be misdiagnosed as an ischemic stroke [10], especially in acute diagnostic settings or in an emergency department (ED). Nevertheless, DS and an acute ischemic stroke might simultaneously occur in the striatum after hyperglycemia; thus, it is easy to misdiagnose DS as an ischemic stroke with a hemorrhage, requiring clinicians to pay more attention to avoid a misinterpretation and delayed treatment [11]. Although DS is a well-known cause of chorea/ballism, most previous reports have not provided a detailed description of the clinical presentation. The occurrence of chorea/ballism is mostly due to a dysfunction of the basal ganglia and subthalamus [12], but other and more common etiologies of chorea/ballism beside DS need to be considered, including cerebrovascular, autoimmune, toxic, malignant, and infectious diseases [5]. Nevertheless, it is reasonable to presume that the ambiguity in the definition of DS and the different terms used to describe it might have contributed to the underestimation of its actual prevalence.

The disorder has been described as predominantly occurring in elderly females with type 2 diabetes mellitus (DM) [9]; the role of the glycated hemoglobin level in predicting DS has been recently highlighted [13]. Although previous case studies proposed that the correction of hyperglycemia usually results in a complete or partial clinical and neuroradiological recovery [1], anti-chorea drugs might be needed in patients unresponsive to glucose control. Chorea may improve with glucose control in 25% of cases, but the majority of patients need other treatments, including GABA-receptor agonists, selective serotonin reuptake inhibitors, and dopamine-depleting agents [14]. However, not only does chorea/ballism substantially impair the activities of daily functions, but also the lack of well-established treatment guidelines may lead to life-threatening complications; for instance, a deep pharmacological sedation such as that induced by typical or atypical anti-psychotic medication may be associated with respiratory failure and a higher mortality rate [15].

Due to its rarity, especially in non-Asian subjects [16], there have been few larger studies to date, and these only focus on the incidence, demographic features, symptoms, and locations of striatal abnormalities of DS [5,17]. One meta-analysis investigated 53 patients [5]; another study analyzed 20 patients [17]. Nevertheless, the discrepancy between the symptoms and neuroimaging, the comparison between CT and MRI, the recovery time of chorea and neuroimaging abnormalities, the effectiveness of different treatments, and the incidence of recurrences as well as variations in the neuropathological features and the disease progression have not yet been explored. For these reasons, the term DS remains ambiguous and occasionally controversial. A recent clinical and radiological study proposed a classification of DS that included symptomatic DS (striatal neuroimaging lesions in association with a clinically evident movement disorder and hyperglycemia), clinically isolated DS (clinically evident movement disorders without striatal changes in neuroimaging), and radiologically isolated DS [18].

Here, we present a DS case mimicking an ischemic stroke and outline a few uncommon/atypical features that might expand the clinical spectrum of DS and gain further insights into its complex pathophysiology. We also report for the first time the findings from parenchymal transcranial sonography (pTCS) performed on DS in the acute phase.

## 2. Case Report

An 86-year-old man presented at an emergency department (ED) because of the occurrence of involuntary jerky movements in his left limbs, which began approximately six hours before. The clinical examination showed isolated choreo-athetotic movements in his left limbs, with fluctuations in the location, frequency, and duration (Suppl. material 1). The rest of the neurological examination was entirely normal. His past medical history included insulin-dependent diabetes, arterial hypertension, chronic ischemic heart disease after a previous myocardial infarction (treated with coronary artery bypass grafting), a biological mitral valve implant, left carotid stenting, prostate hypertrophy, and epilepsy secondary to a previous post-traumatic right hemispheric subdural hematoma. At the time of the examination, he was being treated with ramipril, clopidogrel, levetiracetam, dutasteride, and tamsulosin as well as both fast- and slow-acting insulin; all the medication, including the daily dosages, was not changed during or after his hospitalization.

**Figure 1:**
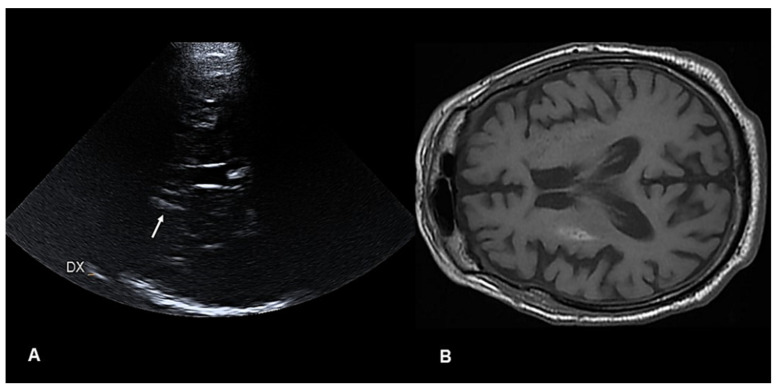
(**A**) (*left panel*) Parenchymal transcranial sonography showing hyperechogenicity in the right (DX) lenticular nucleus (*white arrow*); (**B**) (*right panel*) axial brain magnetic resonance imaging showing a T1-weighted hyperintensity in the right putamen.

The brain CT and angio-CT were negative and a cardiological evaluation showed atrial fibrillation, which was not previously known. The blood glucose level was 569 mg/dL (normal values: 60–100). The search for ketone bodies in the urine was negative. Given that the symptoms occurred after the 4.5 h window for intravenous (IV) thrombolysis and because of the lack of perfusion imaging techniques at the ED, he was treated with 300 mg of IV acetylsalicylic acid. Parenchymal transcranial sonography (pTCS) revealed hyperechogenicity in the right lenticular nucleus (LN). He was admitted to the neurology department, where a second CT was negative. A subsequent MRI showed T1-weighted hyperintensity in the right striatum with negative diffusion-weighted imaging (DWI) and an anatomical correlation with the pTCS finding (Figure 1).

**Figure 2:**
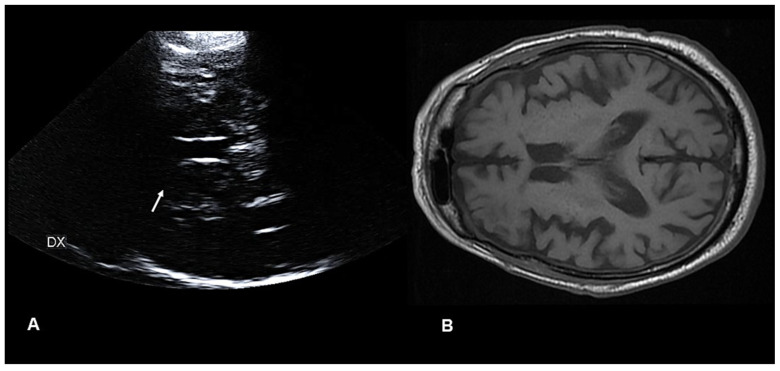
(**A**) (*left panel*) Parenchymal transcranial sonography showing the complete resolution of the hyperechogenicity in the right (DX) lenticular nucleus previously observed (*white arrow*); (**B**) (*right panel*) axial brain magnetic resonance imaging showing normal T1-weighted intensity in the right putamen.

The symptoms were partially responsive to glucose control and haloperidol administration, but persisted during sleep, thus affecting the sleep duration and quality of the patient. As the symptoms were still present both at discharge and after one month, he was shifted to tetrabenazine (25 mg bis in die). The 3-month clinical, pTCS, and MRI follow-up showed a complete resolution of the involuntary movements (Suppl. material 2), as well as of the right LN hyperechogenicity from the pTCS and hyperintensity from the MRI previously observed (Figure 2).

## 3. Discussion

DS is a rare but potentially life-threatening condition, probably underestimated and underdiagnosed in the Western population, with an ambiguous nomenclature and a debated origin. Clinically, hemichorea/hemiballism is the most commonly associated movement disorder in DS and the putamen is the most frequently affected anatomical region, although clinical–radiological discordances are not rare [18,19]. DS typically occurs in patients with long-standing and poorly controlled DM, with average blood glucose and glycated hemoglobin concentrations of 414 mg/dL and 13.1%, respectively [9]; it may even occur after the correction of hyperglycemia [20]. Consistently, almost all DS patients in the literature (96.6%) had type 2 DM, including 1/6 with newly diagnosed diabetes. This implies that DS may be among the first presentations of DM [9]. DS may also, but rarely, be seen in pediatric patients with type 1 diabetes [21].

Regarding the association between DS and ketosis, the large majority of patients (81.7%) with a documented ketone status were not ketotic, including our subject, thus strengthening the term hyperglycemic non-ketotic hemichorea/hemiballism. On the other hand, ketosis in the remaining 18.3% patients implies that the occurrence of DS should not be restricted to non-ketotic patients [9]. The susceptibility of DS to non-ketotic hyperglycemic conditions may arise from the pathophysiology underlying chorea. In a non-ketotic hyperglycemic status, the brain metabolism shifts to the anerobic pathway in the Krebs cycle, leading to the rapid depletion of gamma-aminobutyric acid (GABA); this results in a disinhibition of the basal ganglia and subthalamus that eventually causes the hyperkinetic movements observed in DS. At the other end of the spectrum, in ketosis, GABA can be resynthesized by using acetoacetate produced in the liver to prevent its reduction, thereby explaining the lower occurrence of DS in diabetic ketoacidosis [5,13].

Clinically, chorea in DS mostly involves the unilateral limbs, as in the case presented here, with only 9.7% bilateral involvement [9]. However, unlike the present case (who was fully asymptomatic before the stroke-like onset of chorea), prodromal symptoms are usually reported, including chest pain [22], shoulder pain [23], headaches [24], a gait imbalance [25], hemiparesis [26], lethargy [27], stiffness [28], vertigo [29], dizziness [20,30], confusion [31], and comas [10]. Moreover, the presentation of involuntary movements in DS may vary among patients; they could start abruptly (as described here) or insidiously (from a low to high amplitude) and manifest intermittently or continuously [9]. Cases with chorea progressing from the upper to lower extremities [28,31] are more common than the opposite [32]. In addition, chorea typically worsens during physical or mental stress and, unlike our patient, disappears after sleep. Only two reported cases have shown no suppression of chorea during sleep [33,34]; thus, our patient may possibly be the third case reported. Finally, it should be mentioned that although the majority of DS cases manifested with chorea/hemichorea, in very rare cases the presentation may occur without chorea but with conscious disturbances [31,35], seizures [31,36], limb weakness, dysarthria [37], and dysphagia [38].

Interestingly, although hemichorea hemiballism secondary to DS is becoming increasingly reported, unilateral caudate atrophies as a result of a chronic vascular insufficiency/insult from a backdrop of poorly controlled DM are sparsely described. Recently, a case was reported of a 75-year-old woman with poorly controlled DM who presented with concurrent *epilepsia partialis continua* involving the left side of her face and hemichorea on the right side in the context of non-ketotic hyperglycemia [39]. Neuroimaging revealed a space-occupying lesion suggestive of a low-grade glioma in the right superior frontal cortex and a left-sided caudate atrophy as well. The authors argued that the space-occupying lesion in the motor cortex possibly acted as an inciting factor for the seizures and non-ketotic hyperglycemia further lowered the seizure threshold. On the other hand, the atrophic left caudate might have led to persistent choreiform movements secondary to chronic uncontrolled hyperglycemia, highlighting the need for the strict control of blood glucose and appropriate neuroimaging to rapidly diagnose and prevent further complications [39].

Radiologically, according to the findings of previous reports [5], the most common pattern of striatal anomalies of DS is isolated putamen involvement, followed by a combined caudate nucleus and putamen. The concomitant occurrence of involvement in all three striatal components was noted in over 1/4 of all cases (Table 1) [9]. However, the reason for striatal vulnerability to DS remains unclear. In terms of the body regions affected, despite the highest frequency of extremity involvement in the order of arm–leg, arm–leg–face, and isolated arm, there were two reported cases with isolated facial hemichoreas presenting with oral dyskinesia and grimacing. Of note, no significant association was noted between the body region involved and the location of the striatal anomaly [9].

CT and MRI are the common imaging modalities used to detect the striatal abnormalities of DS. Despite the highly significant correlations between their findings, there was approximately 1/6 of a mismatch and incompatibility between the results [9]. In this regard, we confirmed that MRI was more sensitive to the detection of DS-associated striatal abnormalities as it was also able to demonstrate the striatal lesions in this patient who had negative CT results, although this consideration could only be based on the present subject. Therefore, considering that both CT and MRI can detect changes in different regions of the basal ganglia, CT is still indicated for all patients with negative MRI findings or when MRI is not available or not urgently performed [9].

Regarding the diagnostic sensitivity, Chua et al. [9] compared MRI and CT of 176 patients with DS and found that the sensitivity of the two techniques was 95.3% and 78.9%, respectively. In a very recent case series, MRI and CT examinations at the same period showed the same lesion location but, in terms of scope, the MRI revealed a relatively larger range and it was easier to show a clear edge, thus confirming the greater advantage of MRI than CT in detecting DS [18]. Furthermore, serial MRI, especially when assessed by volumetric analyses, may be particularly useful for DS and its relationship with the clinical features because hemichorea hemiballism may relapse even with the recovery of an euglycemic condition, as previously demonstrated [40].

Although a number of mechanisms can lead to striatal hyperintensity in T1-weighted MRI—including hypertensive hemorrhages, calcification, genetic diseases (e.g., Tay–Sachs’s disease, tuberous sclerosis, neurofibromatosis, and Fahr’s disease), metabolic disorders (e.g., Wilson’s disease, hypoglycemic comas, and chronic hepatic encephalopathy), toxicity (e.g., manganese toxicity and carbon monoxide poisoning), and brain ischemia (e.g., lenticulostriate infarctions, post-cardiac arrest encephalopathy)—the striatal lesions in DS are mostly bilateral [21]. One of the distinctive features of a DS-associated striatal anomaly to differentiate it from a hypertensive hemorrhage is the absence of a mass effect and the sparing of the internal capsule [37], as shown here. This unique imaging finding, when combined with hyperglycemia and chorea, can be considered to be pathognomonic to DS.

From a purely diagnostic perspective, it is worth noting that a differential diagnosis between a stroke and DS may not be possible based on CT alone in the ED; on the other hand, MRI is not always available or cannot urgently be performed in the ED. In this scenario, pTCS hyperechogenicity observed in the right LN, along with normal CT and angio-CT imaging, could help with a differential diagnosis between a metabolic and an ischemic disorder and correctly guide the diagnostic work-up and management, thus also confirming its value in acute settings [38,41].

Over the past 20 years, pTCS has been increasingly recognized as a diagnostic tool in movement disorders. The most known finding in movement disorders is an increase in the echogenicity of the substantia nigra in idiopathic Parkinson’s disease (PD) that allows a reliable diagnosis with a high predictive value. Other sonographic features such as the hyperechogenicity of the LN or CN might help with a differential diagnosis between PD and other movement disorders [42]. Compared with other neuroimaging modalities such as CT and MRI, pTCS is totally safe and non-invasive; it can be easily handled and repeated (e.g., through portable machines) and it has a high resistance to movement artefacts. Notably, in a few disorders, pTCS can detect abnormalities that cannot be displayed or hardly visualized through other imaging modalities, thus often representing an added diagnostic value, especially in emergency settings [43]. Compared with PD, hyperechogenicity of the LN has been reported more frequently in atypical parkinsonism, including the parkinsonian phenotype of a multiple system atrophy or progressive supranuclear palsy [44,45]. Interestingly, LN hyperechogenicity has also been described in patients with Wilson’s disease [46], Creutzfeldt–Jakob disease [47], primary focal dystonia [48], Fahr’s disease [49], and pantothenate kinase-associated neurodegeneration [50]. This would suggest a common, or at least a similar, pathomechanism underlying LN hyperechogenicity, which might lie in an iron–copper–calcium accumulation and the subsequent gliosis [43,46].

To date, a few hypotheses have only tried to explain the pathogenesis of DS and its striatal abnormalities on neuroimages; namely, petechial hemorrhage [51], mineral deposition [14], demyelination [52], and infarction with astrocytosis [12,15]. Researchers initially attributed the striatal anomalies to a petechial hemorrhage, based on the observation of hyperdensity from CT and hyperintensity from MRI, which were strongly suggestive of the presence of a hemorrhage and methemoglobin, respectively [53]. Other earlier studies used different imaging modalities in an attempt to elucidate the cellular function and perfusion status of the affected region. For instance, studies from DWI [54] and susceptibility-weighted MRI [55] suggested hyperviscosity with a cytotoxic edema and the deposition of paramagnetic material, respectively. Positron emission tomography studies demonstrated a marked decrease in glucose metabolism within the lesioned basal ganglia [20,56] and single-photon emission CT mostly revealed hypoperfusions in the corresponding region [57]. A more recent study using magnetic resonance angiography showed straining around the basal ganglia lesions [35].

Unlike radiological studies, neuropathology might shed light onto the pathogenesis of DS. Although myelin destruction may appear as hyperintensity on T1-weighted MRI, there has been no evidence suggesting its presence, at least according to the available neuropathological reports [9]. However, previously documented reactive astrocytosis [8] and abundant gemistocytes in biopsies [12] may explain the striatal hyperintensity from T1-weighted MRI, although not the hyperdensity observed from CT. There are only two pathology reports showing a few calcium deposits [58] or punctuate calcification [15], which could not account for the observed resolution in subsequent neuroimaging studies. In contrast, a microvascular hemorrhage may be an alternative explanation based on a few pathological analyses showing hemosiderin-containing macrophages [58], microhemorrhages [15], extravascular hemosiderin deposits [51], and erythrocyte extravasation [8,59]. Based on these considerations, we hypothesized that iron–calcium-related deposits and the subsequent astrocytosis were among the possible mechanisms underlying the LN hyperechogenicity observed in our patient.

A complete clinical recovery, along with the resolution of imaging findings, is the typical outcome in DS patients. A significant improvement in chorea can be achieved by either an insulin monotherapy or a combination therapy of insulin and a D2-blocker or, in a few cases, spontaneously. Unlike most previously reported cases, however, our patient presented with an acute onset of unilateral irregular choreo-athetotic movements. In this case, pTCS showed a hyperechogenicity of the LN in the acute phase of DS that correlated with MRI and that was no longer evident at both the neurosonological and neuroimaging follow-ups. Although this was based on a single case report—and MRI remains the gold standard diagnostic exam for DS—in the future, pTCS might support the clinical and neuroradiological diagnosis of DS even in the ED, especially when MRI is not available or when it cannot be urgently performed.

Finally, the present report revealed that although chorea can be treated with glucose control only, an anti-chorea medication for the symptom control was needed. There are four main categories of anti-chorea medication: anti-psychotics, GABA-receptor agonists, selective serotonin reuptake inhibitors, and dopamine-depleting agents [13]. Haloperidol is the most common monotherapeutic agent against DS-associated chorea (as used in this case), followed by tetrabenazine, risperidone, and clonazepam. Other anti-chorea drugs include tiapride, quetiapine, pimozide, diazepam, and valproate. Combined regimens have been sporadically documented, and may be indicated for patients with intractable chorea [9].

Prognostically, in a recent descriptive review [9], the lack of a significant difference in the treatment intervals, both between the insulin-control-only group and the additional anti-chorea medication group as well as between the complete response and poor response groups, suggested that the timing of the treatment intervention may not be a critical contributor to the patient outcomes; these appeared to depend more on the severity of the underlying condition. Therefore, the significantly shorter recovery time of the insulin-treatment-only group than that of the additional anti-chorea medication group may reflect a less severe disease in the former compared with the latter. Taking into account the possibility of recurrent chorea even after the resolution of striatal abnormalities [24], the relatively high recurrence rate of close to 20% highlights the need for regular follow-ups [9].

Finally, a few limitations should be acknowledged. First, our study had a single case report-based approach, which precluded any comparison with other patients and alternative treatment options. Second, as with most of the previous studies, we merely searched for the presence of ketone bodies in the urine as a diagnostic tool for confirming or excluding ketosis without mentioning the exact level, so the severity of ketosis could not be determined.

## 4. Conclusions

DS is a rare condition with an overall good prognosis and reversible clinical and neuroimaging findings when promptly diagnosed and managed. DS should be considered as a possible differential diagnosis in patients with the abrupt onset of hyperkinetic movement disorders [60]. With a careful and thoughtful analysis, an accurate diagnosis can be centered, thus sparing the patient unnecessary exams and unfavorable outcomes [61]. In this context, the of use pTCS may be helpful, especially in acute settings or when MRI is not available or cannot be urgently performed.

## Figures and Tables

**Table 1:** Summary of the number of patients (*n*) with locations of striatal abnormalities from brain computed tomography (CT) and magnetic resonance imaging (MRI) (adapted from [9]).

Location	CT (*n* = 126)	MRI (*n* = 153)
Caudate nucleus	1	2
Caudate nucleus and putamen	29	26
Caudate nucleus, putamen, and globus pallidus	30	36
Globus pallidus	0	0
Globus pallidus and caudate nucleus	0	0
Globus pallidus and putamen	5	15
Putamen	35	67
No abnormality	26	7

## Data Availability

Not applicable.

## Supplementary Materials

The following supporting information can be downloaded at: https://www.mdpi.com/article/10.3390/diagnostics12112838/s1, Video S1: Baseline examination of the patient showing isolated choreo-athetotic movements in the left limbs with fluctuations in the location, frequency, and duration. Video S2: Follow-up visit showing the complete resolution of the involuntary movements of the patient.
